# Unlocking the potential of Tregs: innovations in CAR technology

**DOI:** 10.3389/fmolb.2023.1267762

**Published:** 2023-10-12

**Authors:** Christopher J. Requejo Cier, Nicolas Valentini, Caroline Lamarche

**Affiliations:** ^1^ Department of Microbiology, Infectiology and Immunology, Hôpital Maisonneuve-Rosemont Research Institute, Université de Montréal, Montreal, QC, Canada; ^2^ Department of Medicine, Hôpital Maisonneuve-Rosemont Research Institute, Université de Montréal, Montreal, QC, Canada

**Keywords:** regulatory T cells, tolerance, chimeric antigen receptor (CAR), immunotherapy, genetic engineering, cell therapy

## Abstract

Regulatory T cells (Tregs) adoptive immunotherapy is emerging as a viable treatment option for both autoimmune and alloimmune diseases. However, numerous challenges remain, including limitations related to cell number, availability of target-specific cells, stability, purity, homing ability, and safety concerns. To address these challenges, cell engineering strategies have emerged as promising solutions. Indeed, it has become feasible to increase Treg numbers or enhance their stability through Foxp3 overexpression, post-translational modifications, or demethylation of the Treg-specific demethylated region (TSDR). Specificity can be engineered by the addition of chimeric antigen receptors (CARs), with new techniques designed to fine-tune specificity (tandem chimeric antigen receptors, universal chimeric antigen receptors, synNotch chimeric antigen receptors). The introduction of B-cell targeting antibody receptor (BAR) Tregs has paved the way for effective regulation of B cells and plasma cells. In addition, other constructs have emerged to enhance Tregs activation and function, such as optimized chimeric antigen receptors constructs and the use of armour proteins. Chimeric antigen receptor expression can also be better regulated to limit tonic signaling. Furthermore, various opportunities exist for enhancing the homing capabilities of CAR-Tregs to improve therapy outcomes. Many of these genetic modifications have already been explored for conventional CAR-T therapy but need to be further considered for CAR-Tregs therapies. This review highlights innovative CAR-engineering strategies that have the potential to precisely and efficiently manage immune responses in autoimmune diseases and improve transplant outcomes. As these strategies are further explored and optimized, CAR-Treg therapies may emerge as powerful tools for immune intervention.

## Introduction

Autoimmune and alloimmune diseases are major public health challenges. Autoimmune diseases affect millions of individuals worldwide and can lead to severe disability and even death (Jacobson et al., 1997). More than 80 different types of autoimmune diseases have been identified, ranging from lupus and rheumatoid arthritis to type 1 diabetes and multiple sclerosis (Jacobson et al., 1997; Maahs et al., 2010; Wahren-Herlenius and Dörner, 2013). Alloimmune reactions lead to solid organ rejection after a transplantation and graft-versus-host disease (GVHD) after a hematopoietic stem cell transplantation (HCT). Indeed, despite modern immunosuppressive therapy, approximately 8% of kidney transplant recipients will develop acute rejection within 1 year (Lentine et al., 2023), and between 9% and 50% of patients will develop acute GVHD after HCT (Filipovich et al., 2005; Jagasia et al., 2012; Lee et al., 2013).

Immunosuppressive drugs are currently the standard of care for managing these diseases (Sparks, 2019). While these medications can be effective, they also carry significant side effects, including an increased risk of infection, cardiovascular disease, and cancer (Marcén, 2009; Downey, 2016; Salzer et al., 2016). Given these limitations, innovative therapeutic approaches are needed to treat autoimmune diseases and alloimmune responses.

Immunotherapy, an approach that harnesses the ability of the immune system to combat disease, holds great promise for the treatment of autoimmune and alloimmune diseases. Within the complex network of immune cells, regulatory T cells (Tregs) have emerged as key players in the maintenance of immune tolerance (Karim et al., 2005; Waldmann et al., 2006; Sakaguchi et al., 2008; Kassan et al., 2011; Hou et al., 2019). Tregs are a specialized subset of CD4^+^ T lymphocytes characterized by high expression of IL-2Rα (CD25) and the transcription factor forkhead box p3 (Foxp3) (Fontenot et al., 2003; Hori et al., 2003; Khattri et al., 2003), as well as low expression of IL-7Rα (CD127) (Liu et al., 2006). Tregs use a variety of mechanisms to effectively suppress immune responses, including direct cell-to-cell contact and indirect pathways involving the secretion of interleukin-10 (IL-10) and transforming growth factor-beta (TGF-β) secretion (reviewed in (Valentini et al., 2023)).

Preclinical models and early-phase clinical trials using adoptive Treg immunotherapy to promote tolerance have yielded promising results, underscoring the safety and potential effectiveness of this approach (Brunstein et al., 2011; Sarkar et al., 2014; Bluestone et al., 2015; Leclerc and Lamarche, 2021). However, challenges remain in the development and implementation of this therapeutic approach. These challenges arise as early as the isolation and selection of Tregs, further exacerbating their limited availability. Additionally, they affect the potential impact on the specificity, survival, and activation of the acquired Tregs. The use of chimeric antigen receptors (CARs) to redirect the specificity of Tregs has emerged as a promising strategy to improve the efficacy of Treg-based cell therapy (Fransson et al., 2012; Boardman et al., 2017; Noyan et al., 2017; Sicard et al., 2020). CARs are synthetic receptors that redirect cellular specificity independently of major histocompatibility complexes (MHC). CAR-Tregs showed significant potential in preclinical studies in transplantation, autoimmune diseases, and other diseases (Table 1). Further advancements in CAR constructs for Tregs and gene editing technologies hold substantial promise for the development of effective cell therapies. Nevertheless, even though this approach presents a potential solution to the aforementioned challenges, standardization, activation, and the accurate induction of a regulatory phenotype continue to pose hurdles for research teams.

**TABLE 1:** Pre-clinical studies using CAR Tregs.

Disease	Antigen specificity	Main results	References
Transplantation			
GvHD	HLA-A2	Human A2-CAR-Tregs were superior to irrelevant CAR-Tregs to prevent GvHD.	MacDonald et al. (2016)
GvHD and skin transplant	HLA-A2	Human A2-CAR-Tregs prevented A2-expressing human skin graft rejection and prevented GvHD	Noyan et al. (2017)
Skin transplant	HLA-A2	Human A2-CAR-Tregs prevented A2-expressing human skin graft rejection	Boardman et al. (2017)
GvHD and skin transplant	HLA-A2	Human A2-CAR-Tregs prevented A2-expressing human skin graft rejection and prevented GvHD	Dawson et al. (2019)
Skin transplant	HLA-A2	Mouse A2-CAR-Tregs delayed skin rejection, decreased donor-specific antibodies formation and A2-specific B cells formation, but only in unsensitized mice	Sicard et al. (2020)
Heart transplant	HLA-A2	Mouse A2-CAR-Tregs prolonged the survival of heterotopic heart transplants	Wagner et al. (2022)
GvHD, islet and skin	Universal CAR	Mouse mAb-CAR-Tregs could prevent GvHD, prolong survival of islet allografts and secondary skin allografts	Pierini et al. (2017)
GvHD	HLA-A2	Human A2-CAR-Tregs modified to overexpress Foxp3 were stable under proinflammatory conditions and had a survival advantage under IL-2 deprived conditions and could prevent human A2+ PBMC engraftment in humanized mouse model	Henschel et al. (2023)
Autoimmune diseases			
Type 1 diabetes	Insulin B peptide-MHC class II	Mouse CAR-Tregs prevented adoptive transfer diabetes by BDC2.5 T cells in immunodeficient NOD mice and prevented spontaneous diabetes in wild type NOD mice	Spanier et al. (2023)
Type 1 diabetes	Insulin B peptide-MHC class II	Mouse InsB:R3-CAR-Tregs protected against spontaneous diabetes in NOD.CD28−/− mice	Obarorakpor et al. (2023)
Type 1 diabetes	Insulin B	Mouse effector T cells converted to Tregs using insulin-specific Foxp3+ CARs. Long-lived in diabetic mice	Tenspolde et al. (2019)
Colitis	TNP	Mouse TNP-CAR-Tregs improved colitis	Elinav et al. (2008)
			Elinav et al. (2009)
Colitis	CEA	Mouse CEA-CAR-Tregs improved colitis	Blat et al. (2014)
Colitis	Flagellin	Human FliC-CAR-Tregs promoted the establishment of colon-derived epithelial cell monolayers and had a preferential migration to the colon	Boardman et al. (2023)
Multiple sclerosis	MOG (myelin oligodendrocyte glycoprotein)	Engineered mouse MOG-CAR-Tregs suppressed autoimmune encephalomyelitis better than non-specific Tregs	Fransson et al. (2012)
Vitiligo	GD3 (ganglioside D3)	Mouse GD3-CAR Tregs delayed depigmentation compared to untransduced Tregs	Mukhatayev et al. (2020)
Others			
Alzheimer	Beta-Amyloid	Mouse beta-amyloid-CAR-Tregs were suppressive *in vitro*	Saetzler et al. (2023)
Asthma	CEA	Mouse CEA-CAR-Tregs reduced airway hyper-reactivity and diminished eosinophilic airway inflammation	Skuljec et al. (2017)
Hemophilia A	FVIII	Mouse FVIII-CAR-Tregs suppressed the proliferation of FVIII-specific T cells and suppress the recall antibody response	Yoon et al. (2017)
Hemophilia A	FVIII	Mouse CD4^+^ cells lentivirally transduced to express Foxp3 and FVIII-CAR. Promote FVIII tolerance	Fu et al. (2020)

In contrast to effector CAR T cells, CAR constructs designed for Tregs (CAR Tregs) represent a distinct branch within the field of CAR T cell therapies. While effector CAR T cells are primarily engineered to target and eliminate specific disease-related cells, such as cancer cells, by inducing cytotoxicity and releasing pro-inflammatory cytokines (reviewed in (Sterner and Sterner, 2021)), CAR Tregs adopt an immunosuppressive approach by regulating immune responses. Rather than enhancing immune responses, as effector CAR T cells do, CAR Tregs focus on modulating and suppressing immune activity to maintain equilibrium and prevent pathological immune responses. A comprehensive understanding of these distinctions is paramount for tailoring CAR T cell therapies to specific clinical applications and optimizing their outcomes. This review will first address the challenges with the starting material (i.e., selection, purity, quantity), and the later sections will address the challenges associated with CAR constructs.

### Treg selection

**FIGURE 1:**
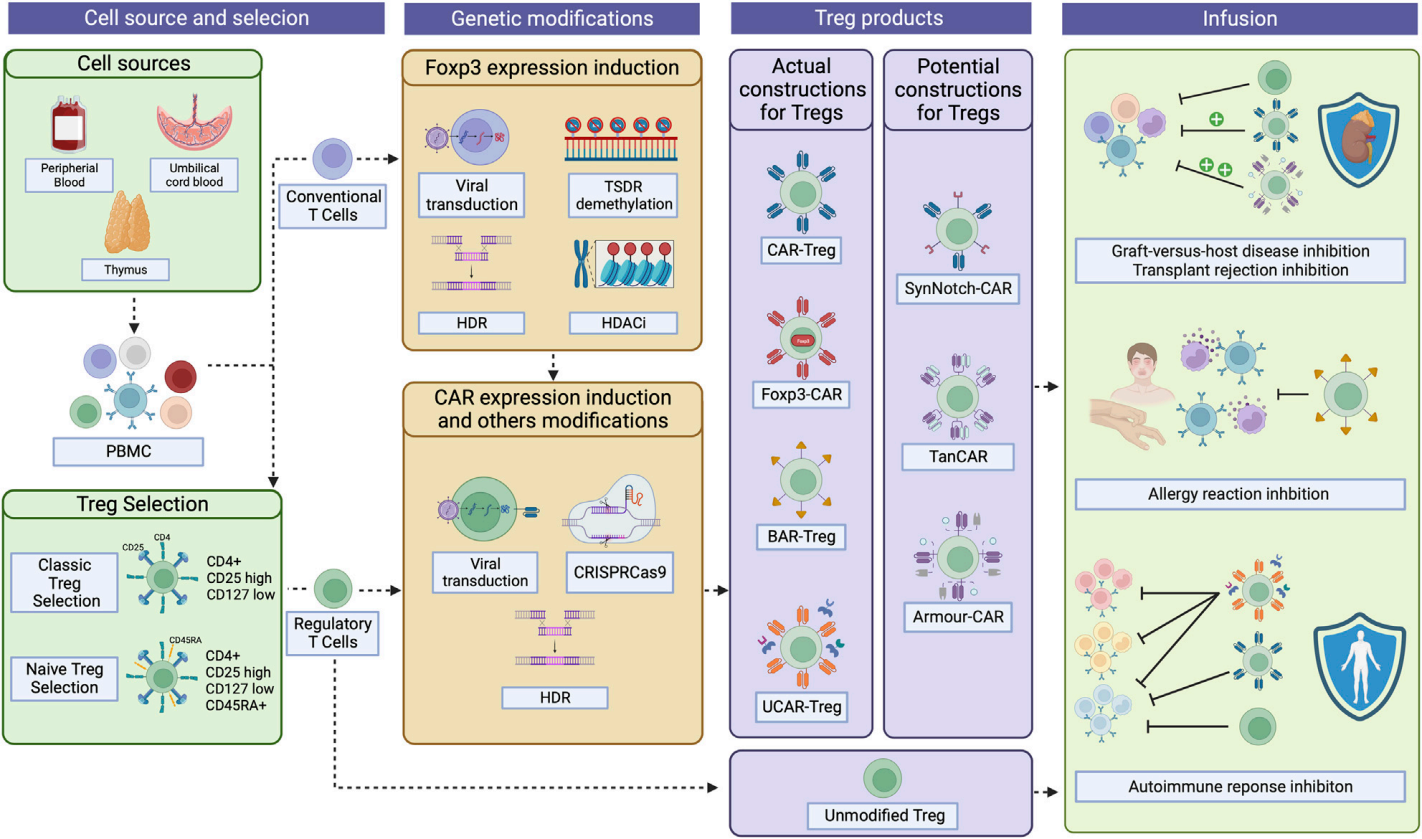
CAR-Treg development workflow. For CAR-Treg development, peripheral blood mononuclear cells (PBMC)s can be retrieved from various sources, including the peripheral blood, umbilical cord blood, or even the thymus. Tregs are then isolated through magnetic enrichment or sorted as CD4^+^CD25^hi^CD127l^ow^ cells. For naïve Tregs, CD45RA^hi^ cells are then selected. Given that obtaining Tregs fromPBMCs can yield limited quantities due to their low proportion, conventional CD4^+^ T cells overexpressing Foxp3 emerge as a possible substrate for CAR-Treg production. Foxp3 can be expressed in those cells after viral transduction, TSDR demethylation, HDR, or the use of HDAC inhibitors. Then, CAR constructs can be integrated into the cells by viral transduction, HDR, or CRISPR-Cas9 technology, among others. To date, four types of CAR constructs have been tested in Tregs: classical CAR, Foxp3-CAR, BAR, and UCAR. Other constructs have, until now, been exclusively tested in effector T cells but holds potential as CAR-Treg therapies: SynNotch-CAR, TanCAR, and Armour-CAR. Then, the desired CAR-Tregs can be infused into patients to treat and/or prevent graft-versus-host disease, transplant rejection, allergies, and autoimmune diseases. CAR, Chimeric Antigen Receptor; TSDR, Treg-specific demethylated region; HDR, Homology-Directed Repair; HDAC, Histone deacetylases; BAR, B cell-targeting Ab receptors; UCAR, Universal CAR; SynNotch, Synthetic Notch CAR; TanCAR, Tandem CAR. Created with BioRender.com.

Tregs are not a homogeneous group of cells; instead, they encompass diverse subpopulations with varying immunosuppressive capabilities, phenotypes, and stability (Figure 1). When using antigen-specific Tregs for cell therapy, ensuring purity and stability is of paramount importance. Indeed, the simultaneous proliferation of antigen-specific conventional T cells alongside Tregs in adoptive immunotherapy could be detrimental, and the maximum ratio of conventional T cells that can be inhibited or contained by Tregs is unknown. One method to enhance cell purity is to select naïve Tregs based on CD45RA expression. This was shown more than 15 years ago by Hoffmann *et al.* (Hoffmann et al., 2006). More recently, this finding has been validated in CAR Tregs (Imura et al., 2020). When compared to CD45RO^+^ Tregs, only CD45RA^+^ CD19 CAR Tregs have demonstrated the ability to maintain substantial levels of Foxp3, Helios, and Ctla-4 expression (Imura et al., 2020). It is worth noting that following activation, conventional T cells can upregulate CD25 expression but will express the CD45RO isoform rather than the CD45RA.

### Exploring new sources of Treg

Tregs, which comprise a mere 5% of CD4^+^ T cells, represent a scarcity that poses a challenge for adoptive immunotherapy. The magnitude of this challenge is amplified in autologous treatments, where certain autoimmune and alloimmune disorders are associated with diminished Treg populations (Demirkiran et al., 2006). Moreover, the administration of immunosuppressive drugs can affect both the quantity and quality of Tregs (Demirkiran et al., 2008). For instance, in the ARTEMIS trial, Tang *et al.* highlight the intricacies involved in Treg manufacturing in liver transplant recipients. In the ARTEMIS trial is a phase I/II trial of autologous donor alloantigen-reactive Tregs in liver transplant recipients, 2–7 years after transplantation, 4/10 were excluded from receiving Treg infusions due to the inability to meet the minimum threshold of 100 × 10^6^ cells. The decrease in donor-specific Treg expansion in liver transplant recipients can be attributed to a lower absolute peripheral blood Treg count compared to participants in other Treg adoptive immunotherapy trials, along with a decrease in the number of donor-reactive Tregs. Furthermore, their analysis suggested a progressive process of Treg activation, exhaustion, senescence, or deletion after liver transplantation (Tang et al., 2022). In an effort to overcome this hurdle, numerous research teams have directed their efforts toward generating novel sources of Tregs, including induced Tregs generation, *in vivo* Treg expansion, or via the genetic reprogramming of conventional CD4^+^ T cells (Figure 1). These innovative approaches aim to augment the pool of available Tregs for therapeutic applications, thereby opening new pathways in the field of adoptive immunotherapy.

#### Induced Treg generation

The first approach involves the induction of Tregs *in vitro*. Indeed, under certain circumstances and factors such as TGF-β and IL-2, naïve CD4^+^ T cells can express Foxp3 and develop a regulatory phenotype (Zheng et al., 2002; Chen et al., 2003). This approach was first tested in HCT recipients in a phase I safety study (MacMillan et al., 2021). Human CD4^+^CD25^−^ conventional T cells were cultured with IL-2, rapamycin and TGF- β with anti-CD3, and artificial antigen-presenting cells to generate induced Tregs, which were then infused into HCT from HLA-identical sibling donors. However, a major concern with induced Tregs is the potential for instability, particularly under inflammatory conditions (Koenecke et al., 2009; Beres et al., 2011). Although patients from this first study did not show an increase in GVHD after infusion, alternative approaches are being developed.

#### *In vivo* Treg expansion

Tregs do not produce IL-2 themselves, but express the high affinity IL-2 receptor CD25. Therefore, the administration of low doses of IL-2 can selectively expand Tregs without conventional T cell activation and expansion (reviewed in (Ye et al., 2018)). However, IL-2 can also activate conventional T cells and NK cells. Consequently, numerous strategies have been developed to selectively increase Tregs and prevent adverse effects, such as Il-2 muteins with greater Treg specificity (Khoryati et al., 2020), anti-IL-2 monoclonal antibodies, or IL-2/anti-IL-2 complexes to modify binding to the IL-2 receptor (Boyman et al., 2006; Polhill et al., 2012; Trotta et al., 2018). Other strategies aim to combine IL-2 with cytokine pathway blockade, such as an anti-IL-6 receptor (Ye et al., 2018), or to modify IL-2 to prolong its half-life, allowing for the use of an ultra-low doses (Bell et al., 2015).

#### Treg generation through Foxp3 overexpression

The first approach to generate Tregs from conventional cells is to overexpress Foxp3 (Figure 1). Indeed, Foxp3 is a master transcription factor for Treg development and function. Sakaguchi *et al.* pioneered this approach, inducing Foxp3 expression in CD25^−^, CD45RO^−^ CD4^+^ lymphocytes by retroviral transduction. The transduced cells exhibited a regulatory phenotype similar to CD25^+^ CD4^+^ Tregs, demonstrated *in vitro* suppressive capabilities, decreased proliferation, cytokine production reduction, and heightened expression of Treg-associated molecules, such as CD25 and CTLA4 (Yagi et al., 2004). While this initial study offers valuable and promising insights into the involvement of Foxp3 in Treg development, it solely showed that the transduced cells displayed a Treg-like phenotype. However, they also exhibited unconventional Treg features, including reduced production of IL-10 (Yagi et al., 2004). Subsequently, Allan *et al.* developed a third-generation lentivirus-based strategy to ectopically express high levels of Foxp3 under the control of the human elongation factor 1α promoter, which does not fluctuate with T cell activation status (Allan et al., 2008). This method facilitated the development of suppressor cells from both naïve and memory CD4^+^ T cells. Nevertheless, despite their demonstrated *in vitro* suppressive capacity, the expected significant production of IL-10 and TGF-β was not observed, suggesting that Foxp3 alone might not be sufficient to induce Treg production (Allan et al., 2008).

Bacchetta *et al.* also tested a third-generation lentivirus-mediated FOXP3 gene transfer in conventional CD4^+^ T cells, confirming its *in vitro* suppressive capacity. Notably, they were the first to test the suppressive ability *in vivo*, demonstrating its effectiveness in a xenogeneic graft-versus-host disease model (xGvHD) (Passerini et al., 2013). However, they did not evaluate IL-10 and TGF-β production in their cells, a notable omission given previous observations. While IL-10 and TGF-β production may not be essential for mediating *in vitro* suppressive function (Allan et al., 2008), their role in the xGvHD model remains unexplored. Finally, Honaker *et al.* used gene editing based on homology-directed repair (HDR) to induce Foxp3 expression (Honaker et al., 2020). The targeted insertion of a robust enhancer-promoter proximal to the first coding exon bypassed epigenetic silencing and ensured stable and strong expression of endogenous Foxp3. The HDR-edited T cells expressed canonical Treg markers and exhibited suppressive capabilities both *in vitro* and *in vivo* (Honaker et al., 2020). However, these cells exhibited lower suppressive potential than normal Tregs and displayed transcriptomic differences from thymic Tregs, such as IKZF2 (Honaker et al., 2020). These observations collectively suggest that Foxp3 expression alone is insufficient to fully recapitulate the Treg function. Given that the transcription factor Helios is implicated in Treg differentiation, stability and suppression, researchers explored the possibility of co-expressing Foxp3 and Helios in conventional cells. However, this co-expression led to significantly reduced cell proliferation and survival, limiting its clinical application (Seng et al., 2020).

Foxp3 overexpression in conventional CD4^+^ T cells has also been combined with CAR expression (Fransson et al., 2012; Martin et al., 2021). This combination enabled the expression of a Treg phenotype, resulting in a reduced IL-2 and TNF-ɑ secretion and increased in IL-10 secretion. Additionally, these cells were shown to be effective in an xGvHD model (Martin et al., 2021) and an autoimmune encephalomyelitis mouse model (Fransson et al., 2012). While this approach is promising, further validation is required before clinical application can be considered.

Strategies aiming at increasing Foxp3 expression may not only serve to convert conventional T cells into Tregs, but also to increase Treg stability. Indeed, Henschel *et al.* developed a specialized CAR vector incorporating Foxp3 into CAR-Tregs (Henschel et al., 2023). They observed significant improvements in both the safety and efficacy of the resulting CAR-Treg product. Notably, in a challenging microenvironment characterized by pro-inflammatory conditions and limited IL-2 availability, Foxp3-CAR-Tregs exhibited sustained Foxp3 expression, surpassing the expression levels observed in control CAR-Tregs. Furthermore, the introduction of additional exogenous Foxp3 expression did not induce any discernible phenotypic alterations or functional impairments, such as cellular exhaustion, loss of Treg characteristics, or aberrant cytokine secretion (Henschel et al., 2023). Overall, these advancements offer potential pathways for the development of more accessible and safe immunotherapies targeting limited Treg numbers. However, further optimization is needed to generate Tregs from conventional CD4^+^ T cells.

#### Treg generation through post-translational modifications

Another method to induce Tregs from conventional CD4^+^ T cells involves the use of epigenetic regulators (Figure 1). Histone/protein deacetylase (HDAC)s can regulate chromatin remodeling, gene expression, and transcription factor expression by removing acetyl groups from histones. This deacetylation process by HDACs typically results in chromatin condensation, restricting access by the transcription machinery and consequently repressing gene expression. Additionally, HDACs function as post-transcriptional modifiers that regulate protein acetylation (reviewed in (Kim and Bae, 2011)). Indeed, HDAC inhibition therapy can induce Foxp3 expression. This therapy increases histone acetylation, which leads to the relaxation of the chromatin structure surrounding the FOXP3 gene. This chromatin decondensation facilitates easier access for transcription factors to interact with the FOXP3 gene, thereby enhancing its transcription and subsequent protein synthesis. Consequently, cells become more proficient at suppressing immune responses and promoting transplant tolerance (Tao et al., 2007; Lucas et al., 2009). There are 18 known mammalian HDACs divided into four classes sometimes with subclasses (I, IIa, IIb, III, IV) and the use of pan-HDAC inhibitors may have various side effects. Therefore, not all HDAC inhibitors promote Treg functions (Wang et al., 2009). Pharmacological targeting and deletion experiments have shown a positive impact on Tregs for HDAC2, HDAC7, HDAC9, HDAC6, HDAC10, HDAC11 and Sirtuin-1. HDAC6, in particular, stands out as the most promising candidate due to the availability of a specific inhibitor and the fact that HDAC6 knockout mice exhibit normal health and behaviour. Hence, the HDAC6 isoform is the primary target for enhancing the immunosuppressive activity of Tregs (de Zoeten et al., 2011; Kalin et al., 2012; Kurohara et al., 2021; Lee et al., 2022). However, HDAC10 could also be of interest, as Wayne Hancock’s team demonstrated an increased suppressive ability of HDAC10−/− mouse Tregs, both *in vitro* and *in vivo* (Dahiya et al., 2020). There is a lot of potential for HDAC inhibitors to induce Tregs *in vivo* (reviewed in (Wang et al., 2018)). However, their use in adoptive immunotherapies and Treg engineering has not yet been explored.

#### Treg generation through Treg-specific demethylation region (TSDR) demethylation

Three conserved non-coding DNA sequence (CNS) elements located at the FOXP3 locus have significant implications for the fate of Tregs. Specifically, CNS1 serves as a TGF-β-sensitive enhancer element crucial for induced Treg generation, CNS2 plays a critical role in stabilizing Foxp3 expression within dividing Treg cells, and CSN3 can modulate Treg frequency in the thymus and periphery but becomes dispensable once Foxp3 is expressed (Zheng et al., 2010). Notably, CSN2, also known as Treg-specific demethylation region (TSDR), exhibits demethylation in stable Treg populations (Feng et al., 2014), in contrast to T cells with transient Foxp3 expression (Floess et al., 2007; Polansky et al., 2008). Recent scientific advances have enabled the selective de-methylation of the TSDR within the endogenous chromatin environment. This epigenetic editing resulted in an increased Foxp3 expression in Jurkat cells (Wilk et al., 2022) and human T cells (Kressler et al., 2020). However, this increase was not associated with phenotypic Treg induction (Kressler et al., 2020). Selective demethylation of the TSDR represents a significant scientific breakthrough. However, it requires further investigation and may need to be combined with other strategies to achieve the desired outcomes.

### Enhancing tregs specificity

**FIGURE 2:**
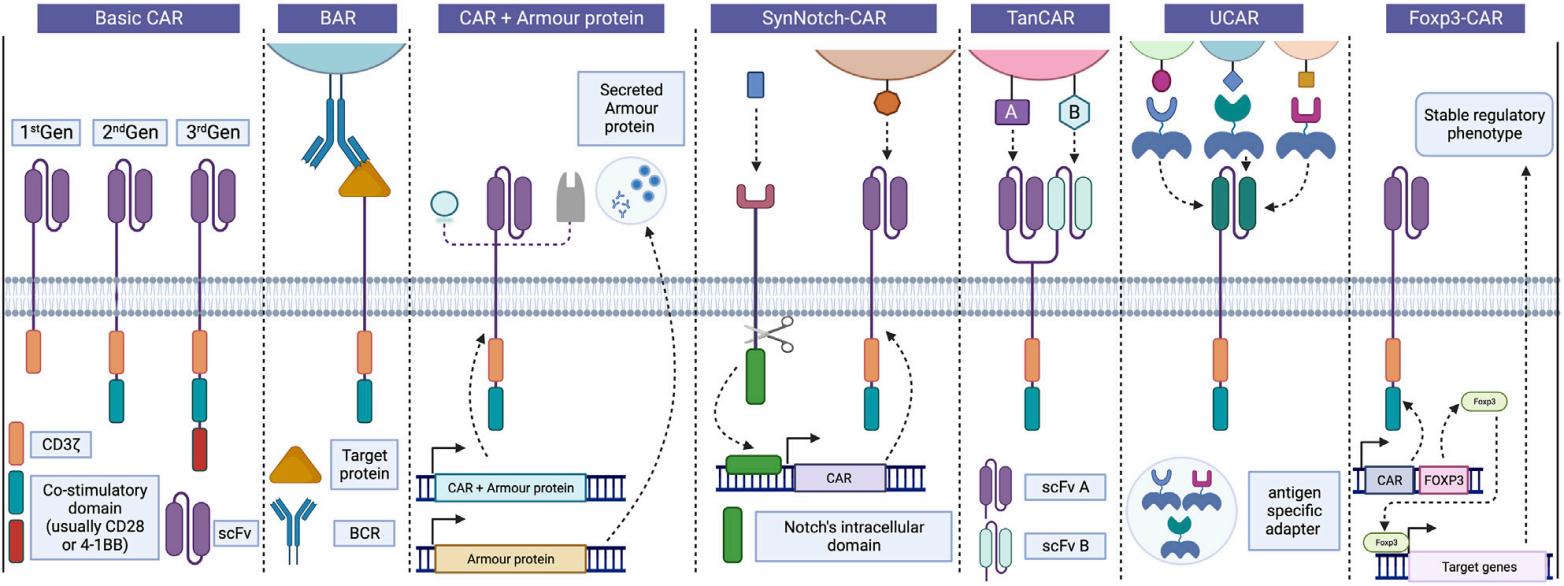
Progress in CAR design and strategies. Basic CARs are divided usually into three generations based on the number of co-stimulatory domains associated with CD3z, CD28 and 4-1BB being the most common. In Tregs, BAR receptors replace the scFv portion for a target protein recognized by the BCR, enabling interaction between Tregs and B cells and inhibiting antibody production. Incorporating “armour proteins” into CAR-T cells improve their function and provides precise control over their activity, enhancing the safety and efficacy of CAR-T therapies in various diseases. These strategies can also be applied to CAR-Tregs to enhance their suppressive capacity and survival. SynNotch CAR strategy employs a positive feedback receptor to regulate CAR-T cell activity, releasing a transcription factor that induces the expression of a CAR targeting a second antigen following recognition of the initial antigen. Bispecific TanCAR cells can recognize two different antigens using distinct scFv portions, but only one of them is required for cell activation, thereby avoiding antigen escape. UCARs enable the binding to target cells through independent and antigen-specific switch modules, allowing for adaptability to different tissue antigens by exchanging adapters. Finally, Foxp3-CAR allows for an increase and stable expression of Foxp3 in Tregs, leading to significant improvements in the safety and efficacy of CAR-Treg products. BAR, B cell-targeting Ab receptors; BCR, B cell receptor; CAR, Chimeric antigen receptor; Foxp3, Forkhead box 3; scFv, Single-chain variable fragment; SynNotch CAR, Synthetic Notch CAR; TanCAR, Tandem CAR; Treg, Regulatory T cells; UCAR, Universal CAR. Created with BioRender.com.

Currently, three clinical trials are registered using monospecific CAR-Tregs in humans (Table 1). However, enhancing the specificity of CAR-Tregs, meaning the number of antigens they can recognize, holds great potential to significantly improve their therapeutic efficacy. The use of monospecific CAR-T effector cells for the treatment of diseases such as cancer has been limited by antigen escape, a phenomenon where the target antigen’s expression is lost, rendering the CAR-T effector cells unable to perform their intended function. In response, several research teams have explored solutions and developed alternative CAR designs (Figure 2), that have shown success in enhancing the effector response (Hegde et al., 2013; Kloss et al., 2013; Roybal et al., 2016; Ruella et al., 2016). Currently, there is no evidence that antigen escape is an concern with CAR-Treg immunotherapies. However, some of the following approaches may prove beneficial.

#### Tandem CAR (TanCAR)

One strategy to expand the diversity of recognized antigens involves the use of bispecific TanCARs, capable of targeting two different antigens (Figure 2). Grada *et al.* developed this novel receptor and demonstrated that TanCARs retain the cytolytic capacity of T cells, even when one of the target molecules is lost. This approach also results in a more controlled response against tumors by recognizing both targets in an animal disease model (Grada et al., 2013). Other studies have adopted a similar strategy, yielding comparable results (Jensen and Riddell, 2014; Zah et al., 2016; Jia et al., 2019). However, this approach has not yet been applied to Tregs.

#### Universal CAR (UCAR)

The application of CAR-Tregs and CAR-T cells is limited by wide range of target antigens dependent on the patient’s condition and by the currently limited catalog of available CAR receptors. To address this challenge, universal CAR platforms (UCAR) were developed (Figure 2). In summary, T cells are engineered with a signaling module that binds to a specific epitope on a switching molecule. The switching module includes an antigen-binding domain and a switching epitope that is recognized by the signaling module (Figure 2). Crosslinking with target cells is thus mediated by these independent antigen-specific switching modules that contain the peptide epitope recognized by the UCAR. These adapters can be easily exchanged to target different tissue antigens, Many UCARs have been engineered and tested in conventional T cells and are reviewed in (Liu et al., 2019). To the best of our knowledge, two groups have tested them in Tregs. Meyer *et al.* tested a CAR that binds antibodies conjugated in the Fc region with FITC. They showed that these CAR-Tregs could prolong mice pancreatic islet allografts and promote tolerance to secondary skin grafts (Batista et al., 2017). Bachmann *et al.* tested a UCAR that recognizes a sequence of 10 amino acids (5B9 tag) of the nuclear protein LA/SS-B (Koristka et al., 2014; Koristka et al., 2018; Kegler et al., 2019), and the transduction procedure itself did not affect the phenotype and activity of the Tregs (Koristka et al., 2014; Koristka et al., 2018). The advantages of UCARs are their high versatility and easy reprogrammability. They could also be safer, as they do not have an effect without the infusion of the switching module. However, as they rely on the infusion of exogenous sequences or epitopes, they require more regulatory examination for safety and efficacy.

#### B cell-targeting Ab receptors (BAR)

The BAR strategy involves replacing the scFv of the CAR with the antigen itself to regulate B-cells and plasma cells that are antigen-specific (Figure 2). It was initially used by Ellebrecht *et al.*, in cytotoxic T cells, to destroy autoantigen-specific hybridoma in a pemphigus vulgaris model (Ellebrecht et al., 2016). This concept was further tested using immunodominant FVIII domains (A2 and C2) to destroy anti-FVIII hybridomas, which are problematic in hemophilia patients (Baaten et al., 2018). The FVIII A2 and C2 domains of BAR-CD8 T cells were assessed *in vitro* (human) and *in vivo* (mouse). The same team then tested their FVIII BAR in Tregs and demonstrated their ability to suppress the memory antibody response in spleen cultures from FVIII-immunized mice *in vitro* and completely prevented the development of anti-FVIII antibodies following FVIII immunization (Zhang et al., 2018). The use of BAR-Tregs also demonstrated promising potential in providing clinical protection against severe allergic reactions. In their study, Abdeladhim *et al.* developed a BAR using ovalbumin (OVA) as the targeted protein (Abdeladhim et al., 2019) and observed a reduction in hypothermia (an indicator of anaphylaxis) following OVA exposure. However, in the *in vivo* setting, no discernible reduction in the circulating levels of OVA-specific IgE was detected (Abdeladhim et al., 2019). Nonetheless, these results have opened the door to the use of BAR-T cells and BAR-Tregs in autoimmune diseases and transplantation.

#### SynNotch CAR

To address the issue of hyperactivity associated with CAR-T cells and the subsequent toxicity concerns, researchers have developed SynNotch receptors incorporating a two-step positive feedback circuit (Figure 2). This circuit comprises a priming signaling receptor devoid of activating-signaling capacity. Upon antigen recognition, these receptors trigger a trans-membrane cleavage, akin to a notch receptor, releasing a transcription factor. This transcription factor then initiates the expression of a CAR targeting another antigen (Figure 2). Both antigens must be present to activate the cell. This technology has been extensively tested in various cancer models using conventional T cells (reviewed in (Stock et al., 2023)) and it holds potential to enhance the effectiveness and safety of CAR-T cell therapies. Thus far it has not been explored in Tregs, but it could prove valuable for targeting combinations of antigens when one or both are widely distributed throughout the body.

### Enhancing activation and function

#### Optimization of the CAR construct

Typically, CARs consist of four domains: an extracellular antigen-binding domain responsible for recognizing the target antigen, a spacer or hinge region that provides the necessary receptor length, a transmembrane domain anchoring the receptor to the plasma membrane, and an intracellular signaling domain that transmits both the first and second co-stimulatory signals (Bridgeman et al., 2010) (Figure 2). Interestingly, different intracellular co-stimulatory domains in second-generation CAR-T cells exert distinct effects on cell differentiation, metabolism, and function. For Tregs, the CD28 co-stimulation domain appears to be the most effective in maintaining Treg function. Initially, Maus *et al.* compared the CD28 co-stimulation domain to 4-1BB, and only CD28 CAR-Tregs demonstrated efficacy *in vivo* (Boroughs et al., 2019). Subsequently, Dawson *et al.* designed a total of 10 anti-HLA-A2 CAR receptors differing in their co-receptor signaling domains. In an xGvHD model, CARs encoding TNFR family co-receptors (OX40, GITR, 4-1BB, and TNFR2) were ineffective in promoting CAR-Tregs function, while CD28-CAR-Tregs exhibited highest effectiveness. PD-1-CAR Tregs displayed ineffective activation, and TNFR2 CARs resulted in a marked increase in TSDR methylation (Dawson et al., 2020). These results were further corroborated by Zuber *et al.*, confirming the negative impact of 4-1BB signaling in Tregs (Lamarthée et al., 2021). These findings offer valuable insights for the future design and optimization of CAR-Treg therapies.

#### Armour proteins

The genetic engineering of CAR-T cells has paved the way for a wide range of innovative approaches aimed at enhancing their efficacy and anti-tumoral function. One particularly intriguing strategy involves the incorporation of “armour” proteins through modifications in CAR genes. These proteins, whether secreted or expressed on the cell surface, possess the potential to augment T cell activity and positively influence the tumor microenvironment (Figure 2). By finely tuning each component of CAR genetic constructs, precise control can be exerted over the functionality, capacity, and safety of the resulting CAR-T cells.

An example of such advancement can be found in Cherkassky *et al.*‘s study, which developed anti-mesothelin CAR-T cells transduced with a truncated dominant-negative PD-1 receptor. This receptor, devoid of intracellular signaling domains, binds to PD-L1 but fails to transmit inhibitory signals. Consequently, it enables the CAR-T cells to resist PD-L1-induced exhaustion, thereby prolonging survival in xenograft models of pleural mesotheliomas (Cherkassky et al., 2016). Another notable study by Yeku *et al.* focused on the design of CAR-T cells capable of constitutive secretion of IL-12. In a model of ovarian peritoneal carcinomatosis, these CAR-T cells exhibited enhanced expansion and greater anti-tumoral efficacy (Yeku et al., 2017).

In the field of adoptive immunotherapy, the use of armour proteins holds great potential for enhancing the suppressive and survival capabilities of CAR-Tregs. Employing secreted armour proteins such as IL-2 could potentially improve proliferation and survival, while TGF-β or IL-10 (Mohseni et al., 2021) could foster a more immunosuppressive environment. However, it should be noted that IL-10 overexpression alone may not suffice to maintain tolerance if cells lose other immunosuppressive abilities (Rana et al., 2021). The co-expression of membrane molecules like Pd-1 and Ctla-4 with the CAR could enhance contact-mediated inhibitory capacities. Furthermore, the expression of transgenic receptors can also be combined with small-molecule therapeutics to promote efficacy and/or survival. For example, the incorporation of an orthogonal IL-2 receptor, selectively responsive to orthogonal IL-2 injection yet retaining retains native IL-2 signalling through STAT5, can facilitate the selective expansion of the transduced Tregs (Sasaki et al., 2014; Hirai et al., 2021). Given these significant advancements, CAR-Tregs designed with armour proteins emerge as a promising immunotherapy modality applicable to various disorders.

### Fine-tuning CAR expression

In most studies, CARs have been introduced into Tregs through lentiviral or retroviral transduction. This method, however, leads to continuous expression of the endogenous TCR and poses the risk of random integration of the CAR construct into the genome, resulting in varying levels of CAR expression, transcriptional silencing, or unintended disruption of crucial genes. Strong expression can also lead to clustering of the CARs, leading to ligand-independent tonic signaling, and consequently, to CAR-T cell (Long et al., 2015) and Treg exhaustion (Lamarche et al., 2023).

To circumvent these consequences, Eyquem *et al.* devised a precise integration system employing CRISPR-Cas9 to introduce an anti-CD19 CAR at the constant locus of the T cell receptor alpha (TRAC) in effector T cells. Their strategy involved designing a gRNA targeting the 5′end of the first exon of TRAC and employing an adeno-associated virus (AAV)-based repair vector encoding a self-cleaving P2A peptide followed by the CAR-CD19 cDNA. The resulting CAR-T cells not only demonstrated uniform CAR-CD19 expression, but also exhibited a reduction in tonic signaling, exhaustion and an increase in antitumour efficacy (Eyquem et al., 2017). TRAC-CAR-T cells also surpassed conventionally edited CAR-T cells in an acute lymphoblastic leukemia murine model. Additionally, the insertion at the TRAC locus prevented tonic signaling and facilitated effective CAR internalization and subsequent re-expression following single or repeated antigen exposure, effectively delaying effector T cell differentiation and exhaustion (Eyquem et al., 2017).

Building upon this approach, Muller *et al.* generated human anti-HLA-A2 CAR-Tregs into the TRAC locus after TCR expression elimination. They employed CRISPR-Cas9 to eliminate TCR expression and achieved the introduction of the anti-HLA-A2 CAR construct through direct integration into the TRAC locus using homology-directed repair (HDR). The resulting cells demonstrated uniform CAR expression, retained their phenotype (Foxp3^+^, Helios^+^), and exhibited A2-specific suppressive functionality *in vitro* and *in vivo*. These cells demonstrated a delay in GvHD development, exclusively in the presence of HLA-A2, whether expressed by simultaneously injected peripheral blood mononuclear cells or the recipient mice themselves. These outcomes effectively illustrated that these genetically engineered CAR-Tregs display antigen-dependent *in vivo* suppression, independent of TCR expression (Muller et al., 2021).

Another approach is to create an alternative TCR fusion construct (TRuC), which fuses the scFv to the N terminus of the TCRε subunit (Baeuerle et al., 2019; Ding et al., 2023; Lesch et al., 2023). This approach can limit the CAR density on the cell surface and better replicates physiological TCR signaling. It has been applied to Tregs and has demonstrated great immunosuppressive potential (Rana et al., 2021).

### Increasing Treg homing

Shaping Tregs for better homing abilities to specific inflammation sites has been a topic of interest since the early 2000s. Studies investigating homing patterns of Tregs using UV irradiation showed migration to lymph nodes upon injection (Wilk et al., 2022). The induction of conventional T cells could then be reduced as T-cell priming occurs within the lymph nodes. To efficiently suppress inflammation in the periphery, local injection of Tregs could circumvent this limitation for specific external area-targeted immunotherapy (Schwarz et al., 2004). The alternative is to use integrin or chemokine expression to facilitate specific homing (reviewed in (Lamarche and Levings, 2018)). Their potential in homing-target specific-CARs have yet to be fully established, but preclinical studies on CD4 T cells have lead the way for some of our current models (Table 2).

**TABLE 2:** Homing markers on CD4 T cells for increased target-specificity.

Homing marker (T cell receptor)	Binding molecule (tissue target)	Organism	References
CCR1 (CD191)	CCL3, CCL5, CCL7, CCL16 (induced by inflammation)	mammals	Neote et al. (1993)
CCR2 (CD192)	CCL2, CCL7, CCL12 (induced by inflammation)	most animals	Boring et al. (1997)
CCR3 (CD193)	CCL5, CCL7, CCL8 (induced by inflammation)	mammals	Gerber et al. (1997)
CCR4 (CD194)	CCL17, CCL22 (induced by inflammation)	most animals	Imai et al. (1999)
CCR5 (CD195)	CCL3, CCL4, CCL5 (induced by inflammation)	most animals	Reviewed in (Oppermann, 2004)
CCR6 (CD196)	CCL20 (skin, intestine)	most animals	Cook et al. (2000)
CCR7 (CD197)	CCL19, CCL21 (lymphoid organs)	most animals	Förster et al. (1999)
CCR8 (CD198)	CCL1 (induced by inflammation)	mammals, birds, fish	Schaerli et al. (2004)
CCR9 (CD199)	CCL25 (small intestine)	most animals	Uehara et al. (2002)
CCR10 (GPR2)	CCL27 (skin, increased with inflammation)	mammals, birds, reptiles	Homey et al. (2002)
CD44 (Hermes-1)	Hyaluronic acid, E-selectin (tissue injury)	most animals	Horst et al. (1990)
CD62L (SELL)	α-Actinin, CaM, ERM proteins, PKC and others (vaculature and lymph nodes entry)	most animals	Reviewed in (Ivetic et al., 2019)
CX3CR1 (V28)	CX3CL1 (Fractalkine) (migration and survival of some CD4 subsets)	mammals, birds, reptiles	Yadav et al. (2011)
CXCR1 (IL8RA)	CXCL6 (GCP2), CXCL8 (IL-8) (inflammation, tumors)	mammals, fish	Francis et al. (2004)
CXCR2 (IL8RB)	CXCL8 (IL-8), CXCL1, 2, 3, 5, 6, 7 (inflammation, tumors)	mammals	Jin et al. (2019a)
CXCR3 (GPR9)	CXCL9, CXCL10 (IP-10) (inflammation sites mediated by IFNγ)	mammals, reptiles, fish	Reviewed in Groom and Luster (2011)
CXCR4 (Fusin)	CXCL12 (SDF-1) (marrow microenvironment)	most animals	Reviewed in Burger and Bürkle (2007)
CXCR5 (MDR15)	CXCL13 (BLC) (migration to lymphoid organs, mostly for B cells)	most animals	Reviewed in Kazanietz et al. (2019)
CXCR6 (Bonzo)	CXCL16 (Th1 inflammation)	mammals, reptiles, amphibians	Agostini et al. (2005)
ESL-1 (CFR-1)	E-selectin (CD62E) (inflamed venules)	most animals	Reviewed in Hidalgo et al. (2007)
PSGL-1 (CD162)	P-, E−, L-selectin, VISTA, CCL19, CCL21 and others (inflammation, tumors)	mammals and reptiles	Reviewed in DeRogatis et al. (2021)
ST2 (IL1RL1)	IL-33 (Th2 inflammation)	most animals	Shimizu et al. (2005)
α4β1 (VLA-4)	VCAM-1 (bone marrow and sites of inflammation)	most animals	Reviewed in Chavakis (2012)
α4β7 (LPAM-1)	MAdCAM-1 (mesenteric LN, small intestine, sites of inflammation)	most animals	Berlin et al. (1993)
αEβ7 (CD103)	E-cadherin (epithelia)	mammals, reptiles, fish	Higgins et al. (1998)
αLβ2 (LFA-1)	ICAM-1 (postcapillary venules, increased with inflammation)	mammals, reptiles, fish	Reviewed in Yusuf-Makagiansar et al. (2002)
αvβ6 (ITGB6)	fibronectin, tenascin-C, LAP of TGF-β1, others (solid tumors, inflamed tissues)	most animals	Reviewed in Bandyopadhyay and Raghavan, (2009)

It should also be considered that antigen-specific Tregs inherently exhibit more efficient homing abilities than expanded Tregs generated from polyclonal pools (Lam et al., 2017). The homing abilities of CAR-Tregs surpass those of TCR-engineered Tregs because they do not rely on MHC-presentation and are less dependent on IL-2 (as reviewed in (Campbell, 2015)). Adding supplemental factors to these engineering and expansion protocols for CAR-Tregs, such as RA, transduced KLF2, or other Th-mediated response elements, may lead to a newer generation of Tregs that are even more tailored and efficient in their immunosuppressive capabilities and specifically suited for diverse contexts.

Studies in conventional CAR-T cell therapy have demonstrated that chemokine-specific CARs can more efficiently inhibit the growth of solid and immunosuppressive cancers. Human solid tumors xenograft models have been evaluated for chemokines such as CXCL1-2. The intra-tumoral presence of CAR-T cells increased when modified for CXCR1-2 surface expression (Jin et al., 2019b). Transduction of CXCR2 in CAR-T cells targeting αvβ6, an integrin strongly expressed in pancreatic cancer, controlled tumor growth in a xenograft mouse model (Whilding et al., 2019). Known mechanisms of Treg homing in breast cancer have also been studied such as the CCL1-CCR8 axis. To improve trafficking and resistance to immunosuppression, CCR8-modified CAR-T cells were transduced for a TGFβ-insensitive receptor (DNRII) and showed consistent tumor regression compared to CCR8-only expressing CAR-T cells (Cadilha et al., 2021).

Such CAR modifications in Tregs could lead to inflammation-specific immunosuppression. Expression of α4β7-integrin, CCR9 and CCR6 in Foxp3^+^ Tregs enhances recruitment to sites of intestinal inflammation (Elias et al., 2008; Menning et al., 2010). CXCR3 in Tregs, such as found in Tbet^+^Foxp3^+^ T helper (Th)1-Tregs, improves migration towards CXCL9, 10 and 11 (Hoeppli et al., 2019). The transduction of these chemokine receptors in CAR-Tregs requires further study to evaluate their efficiency. For a more Th2-like mediated response in humoral dysregulations, IL-33 receptor (ST2) and CCR4, which have been shown to be involved in tissue-resident Treg maintenance and better graft retention, could also be considered for chemokine-modified CAR-Tregs (Vasanthakumar et al., 2015). The expression of CCR2, CCR5 and CCR7 expression on CARs Tregs may reduce graft rejection due to better migration to lymph nodes (Zhang et al., 2009). The stability of these newly engineered CAR-Tregs under *in vitro* and *in vivo* inflammatory expansion conditions requires further investigation. However, it is worth noting that natural Tregs have demonstrated the ability to maintain their suppressive phenotype (Dijke et al., 2016).

### Safety

CAR-Tregs are likely to be safer than conventional CAR-T therapies because they should not cause problems such as tumor lysis syndrome, hematotoxicity and cytokine release syndrome. Theoretically, there is a risk of off-target effects resulting from their immunosuppressive effects, such as an increased risk of infection or cancer. However, all phase I/II clinical studies using Treg adoptive immunotherapy have demonstrated the safety of this therapy, with no evidence of increased risk of infection or cancer (Santegoets et al., 2015; Abhishek et al., 2023). Another potential concern would be the contamination of Treg products with conventional T cells, which could exacerbate autoimmune or alloimmune diseases. In the event of an infection or cancer, elimination of Tregs with immunosuppressive drugs could worsen the condition. Alternatively, a reversible or permanent “OFF” switch could be used to eliminate CAR-Tregs without disturbing the immune system. As the first clinical trials of CAR-Treg therapy in humans are currently underway, their safety has yet to be demonstrated (Table 3).

**TABLE 3:** Registered clinical trials using CAR-Treg adoptive immunotherapy.

Trial name	Population	CAR characteristics	Clincaltrial.gov ID
STeadfast	Living donor renal transplant recipients	HLA-A2 CAR-Tregs, autologous	NCT04817774 (protocol in Schreeb et al. (2022), commented in Lamarche and Maltzman (2022))
LIBERATE	Liver transplant recipients	HLA-A2 CAR-Tregs, autologous	NCT05234190
Allogeneic CD6-CAR Tregs for the treatment of patients with chronic graft versus host disease after allogeneic hematopoietic cell transplantation	Allogenieic hematopoietic cell transplantation recipients, for the treatment of chronic graft-versus-host disease	CD6-CAR-Tregs, allogeneic (donor-derived)	NCT05993611
CAR19 Regulatory T Cells (CAR19-tTreg) in Adults With Relapsed/Refractory CD19^+^ B Acute Lymphocytic Leukemia	Adults with relapsed/refractory (R/R) CD19^+^ B Acute Lymphocytic Leukemia (B-ALL)	CAR19-Treg, allogeneic	NCT05114837

One approach that has been developed for conventional CAR-T cell therapy involves the use of kinase inhibitors, such as dasatinib, to induce a functional “OFF” state (reviewed in (Stock et al., 2023)). However, this approach has not been studied in Tregs, and the differential impact on Tregs compared to conventional T cells has not been assessed. This approach could also be detrimental if it leads to immune hyporesponsiveness due to increased off-target effects arose.

Another strategy involves the use of enzyme inhibitors, which can reversibly lead to CAR degradation. This requires the addition of a bromodomain or protease domain on the CAR, which can be modulated using a proteolysis-targeting chimera compound or a protease inhibitor (reviewed in (Stock et al., 2023)). None of these strategies have been studied in CAR-Tregs thus far.

Permanent elimination of CAR-T cells can be achieved by integrating suicide genes into these cells. For example, metabolic suicide gene systems can be employed with a non-toxic prodrug therapy administered systematically. Genetically-modified cells possess the gene necessary to convert the prodrug into a toxic compound (reviewed in (Stock et al., 2023)). Alternatively, a chemical inducer of dimerization could also be administered to induce dimerization and activation of components of the apoptotic pathway (reviewed in (Stock et al., 2023)). Cells can also be engineered to co-express a target marker alongside the CAR. These options include CD52, CD20, the truncated epidermal growth factor receptor (EGFRt), or an EGFR-FOLR1 fusion receptor. However, the impact of administering anti-CD52, anti-CD20 or anti-EGFR antibodies to already immunocompromised patients must be carefully considered. In the case of an infection or cancer, these drugs might even have detrimental effects.

## Conclusion

The development of advanced generations of CARs, such as TanCARs, UCARs, SynNotch CARs, or BARs, holds great promise for CAR-Treg therapy by overcoming challenges related to immunogenicity and production limitations. Furthermore, the incorporation of armour proteins and safety mechanisms like suicide genes, as well as enhancements of homing abilities through chemokine receptors, have the potential to revolutionize the targeting precision and stability of CAR-Tregs. The use of these innovative constructs brings us closer to achieving precise, safe, and efficient treatments for autoimmune and alloimmune diseases. However, further progress requires a rigorous evaluation of these novel CAR-Tregs in preclinical models prior to their introduction into clinical trials.
